# Improved generation of rat gene knockouts by target-selected mutagenesis in mismatch repair-deficient animals

**DOI:** 10.1186/1471-2164-9-460

**Published:** 2008-10-07

**Authors:** Ruben van Boxtel, Pim W Toonen, Mark Verheul, Henk S van Roekel, Isaac J Nijman, Victor Guryev, Edwin Cuppen

**Affiliations:** 1 Hubrecht Institute for Developmental Biology and Stem Cell Research, Cancer Genomics Center, Royal Netherlands Academy of Sciences, Utrecht, The Netherlands; 2 University Medical Center Utrecht, Uppsalalaan 8, 3584 CT Utrecht, The Netherlands

## Abstract

**Background:**

The laboratory rat *(Rattus norvegicus) *is one of the preferred model organisms in physiological and pharmacological research, although the availability of specific genetic models, especially gene knockouts, is limited. *N*-ethyl-*N*-nitrosourea (ENU)-driven target-selected mutagenesis is currently the most successful method in rats, although it is still very laborious and expensive.

**Results:**

As ENU-induced DNA damage is normally recognized by the mismatch repair (MMR) system, we hypothesized that the effectiveness of the target-selected mutagenesis approach could be improved by using a MMR-deficient genetic background. Indeed, Msh6 knockout rats were found to be more sensitive to ENU treatment and the germ line mutation rate was boosted more than two-fold to 1 mutation per 585 kb. In addition, the molecular mutation spectrum was found to be changed in favor of generating knockout-type alleles by ~20%, resulting in an overall increase in efficiency of ~2.5 fold. The improved effectiveness was demonstrated by high throughput mutation discovery in 70 Mb of sequence in a set of only 310 mutant F1 rats. This resulted in the identification of 89 mutations of which four introduced a premature stopcodon and 64 resulted in amino acid changes.

**Conclusion:**

Taken together, we show that the use of a MMR-deficient background considerably improves ENU-driven target-selected mutagenesis in the rat, thereby reducing animal use as well as screening costs. The use of a mismatch repair-deficient genetic background for improving mutagenesis and target-selected knockout efficiency is in principle applicable to any organism of interest.

## Background

The rat is one of the most widely used model organism in biomedical research and has proven to be a powerful tool for linking physiology and pathology to the genome [1,2]. Selective breeding and characterization of strains, mimicking complex human diseases have led to hundreds of useful models. However, identification of the underlying causative polymorphisms and genes has shown to be difficult. An alternative approach in elucidating specific gene functions is by introducing targeted genetic modifications. Gene knockout technology using homologous recombination in embryonic stem cells has proven to be extremely powerful for this [3]. However, due to the lack of pluripotent embryonic stem cells for the rat, ENU-driven target-selected mutagenesis, also known as TILLING [4] has been one of the most successful methods for generating knockouts in the rat [5,6]. This approach does not require any special cell lines and/or advanced oocyte or embryo manipulation as male animals are injected with the alkylating agent ENU, which very efficiently introduces random point mutations in the DNA of spermatogonial stem cells. Upon crosses with untreated female animals, an F1 generation is established in which each individual carries different random heterozygous point mutations in its genome. The DNA of these animals is subsequently screened in pre-selected genes of interest with the goal to identify mutations that affect protein function, e.g. by introducing a premature stopcodon, by changing a consensus splice site residue, or by mutating critical (conserved) amino acids.

The overall efficiency of this method depends essentially on the mutagenicity of ENU, which was found to be both strain and dose dependent [5-7]. The maximum ENU-induced mutation rate in rats is approximately one point mutation every 1.25 – 1.5 Mb for Wistar rats treated with 35–40 mg ENU per kg bodyweight [5], which is similar to the highest mutation frequency that can be obtained in mice [8-12]. Higher doses of ENU result in sterility of the animals and at much higher doses (>120 mg/kg for rats) in lethality. However, in Arabidopsis and *C. elegans *chemically induced (EMS) point mutation frequencies are as high as 1 in 100 kb [13,14] and in zebrafish ENU-induced frequencies of 1 in 150–250 kb can be obtained routinely [15] (E.C., unpublished results), suggesting that the maximum mutation load in a vertebrate genome that is compatible with viability, is much higher than what is currently reached in both rat and mouse. Although about 10 rat knockouts were successfully generated by ENU-driven target-selected mutagenesis [5,6,16] the relatively low mutation frequency makes the target-selected mutagenesis procedure laborious and costly.

We hypothesized that the efficiency of the ENU-driven target-selected mutagenesis approach could potentially be increased in a MMR-deficient genetic background, as this system is involved in the response to and repair of ENU-induced genetic damage [17]. Single nucleotide mismatches, as well as small insertion and deletion loops (IDLs), are recognized by MutSα heterodimer, which consists of Msh2 and Msh6 [18]. Because Msh2 also functions in another heterodimer with Msh3, which recognizes larger IDLs, Msh6-deficiency is in principle best suited to test the idea if the ENU mutagenesis efficiency can be increased in a MMR-deficient background. The MutSα subunit has already been implicated in the recognition of different forms of DNA damage caused by genotoxic agents [19]. Indeed, cell lines deficient in MutSα function that were treated with different alkylating agents, including ENU, showed a higher survival and increased mutation rate [17].

A rat carrying a premature stopcodon in the *Msh6 *gene has been identified in a previous knockout screen in our lab [5]. This mutation was shown to result in a complete loss of function [20] and, as a result, these animals show genetic instability as indicated by microsatellite instability (MSI), an elevated spontaneous germ line point mutation rate and tumorigenesis [20].

Here, we show that Msh6-deficiency improves the efficiency of the ENU-driven target-selected mutagenesis procedure in the rat by ~2.5-fold. This does not only result in a vast decrease of the number of animals needed, but equally reduces screening costs.

## Results

### Effect of ENU in Msh6 knockout rat

The efficiency of ENU is both strain and dosage-dependent, which is reflected by very large differences in mutation frequency in different mouse [7] and rat [5,6] inbred strains and by a higher mutagenicity after three weekly administrations of low doses of the mutagen as compared to a single high-dose injection [7]. Higher ENU concentrations not only increase the mutation frequency, but also affect fertility [5,21]. To determine the optimal concentration in the *msh6*^-/- ^rat (which is in Wistar background), we performed a dose-response experiment and determined the impact of the mutagen on fertility. At 12 weeks of age *msh6*^-/- ^males were treated with 3 weekly doses of ENU ranging between 1 and 35 mg/kg bodyweight and fertility was monitored after a full cycle of spermatogenesis (~60–70 days). At concentrations of 35 and 30 mg/kg, respectively 1 out of 8 and 5 out of 8 males were fertile (Table 1). Previous studies showed that wild type Wistar rats are less sensitive to ENU as 6 out of 10 and 10 out of 10 rats were fertile at 40 and 35 mg/kg ENU, respectively [5].

**Table 1:** ENU-induced mutation frequencies

**Genotype/Dose^a^**	**Msh6^-/-^3 × 25**	**Msh6^-/-^3 × 30**	**Msh6^-/-^3 × 35**	**Total**	**WT Control 3 × 40^b^**
**# Males injected**	6	8	8	24	10
**# Fertile males^c^**	N/D	5	1		6
**# Pups for screening**	16	291	3	310	362
**# Bases screened**	3.6 × 10^6^	69.7 × 10^6^	0.7 × 10^6^	74.0 × 10^6^	37.3 × 10^6^
**# Mutations**					
**- nonsense**	0	4	0	4	0
**- missense**	4	59	1	64	17
**- silent**	0	16	0	16	7
**- non-coding**	0	5	0	5	6
**- total**	4	84	1	89	30
**Mutation Frequencies (bp**^-1^)	1.12 × 10^-6^	1.21 × 10^-6^	1.38 × 10^-6^		8.05 × 10^-7^
**Mutation Rates (bp)**	1 in 8.93 × 10^5^	1 in 8.29 × 10^5^	1 in 7.22 × 10^5^		1 in 12.4 × 10^5^

^a^Dose is indicated as mg of ENU per kg bodyweight (three weekly injections)

^b^WT (Wild Type) control 3 × 40 mg/kg measurements were adapted from [5] and represent the highest ENU-induced mutation frequency in rats

^c^Fertility is measured by the presence of at least one litter within 10 weeks after the last injection.

Sterility was confirmed by histological examination (N/D = not determined)

**Figure 1:**
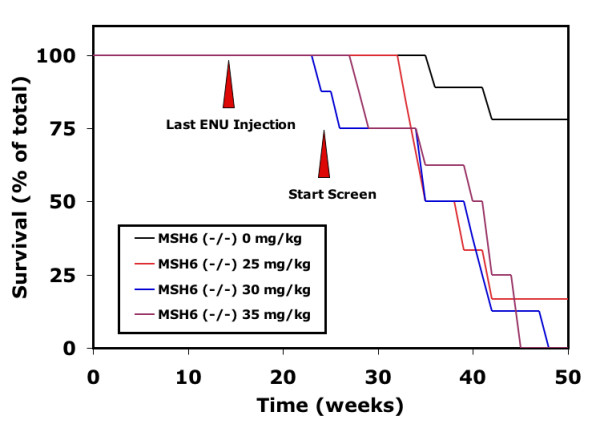
**Effect of ENU on the survival of *msh6*^-/- ^males**. *Msh6*^-/- ^male rats show an increased mortality after treatment with different concentrations of ENU compared to untreated *msh6*^-/- ^male rats. Survival of untreated and ENU-treated wild type rats is 100% in the indicated time-period (data not shown). Red arrowheads indicate the time points of the last ENU injection and the start of mating for F1 progeny that can be screened for mutations without risk for chimaeric progeny.

We also found that ENU affects the survival of *msh6*^-/- ^rats. Untreated *msh6*^-/- ^rats show a median survival of 60 weeks [20], while ENU treatment decreased survival of mutant males to an average mean of 37 ± 3 weeks (Figure 1). This decrease in lifespan was found to be due to tumor development, mainly lymphomas (data not shown). It is crucial for performing ENU target-selected mutagenesis screens that animals are viable and healthy for at least 26 weeks to allow for mutagenesis and subsequent breeding. In previous studies, no reduction in lifespan was observed in both 40 mg/kg (10 out of 10 survived) and 35 mg/kg (9 out of 10 survived) ENU-treated wild type males until 1.5 years of age (E.C., unpublished data).

### Mutation screening procedure

We have developed an automated, high-throughput and cost-effective dideoxy-based DNA resequencing protocol (~0.15 € for chemicals and disposables per sample) for the discovery of ENU-induced point mutations [5]. To reduce the costs of the knockout procedure further, we now established an efficient breeding and screening schedule in which the offspring of mutagenized animals are screened for the presence of interesting mutations before they are weaned (3 weeks of age). This rolling-circle model significantly reduces the amount of animal space needed.

At two weeks of age tissue samples from uniquely tagged F1 offspring were taken and DNA was isolated. Subsequently, DNA samples were screened for 768 pre-selected amplicons by nested PCR amplification and sequencing using two sets of 384 wells plates, containing pre-gridded primers. Resequencing data for the same amplicons from different pups were automatically processed by PolyPhred [22] as well as semi-manually inspected using in-house developed software. All candidate mutations were verified in independent PCR and sequencing reactions and within one week interesting mutants could be selected and weaned.

### Increased ENU mutagenicity in Msh6-deficient background

The highest ENU dose resulting in more than 25% fertile males after a full cycle of spermatogenesis was found to be 3 × 30 mg/kg for the *msh6*^-/- ^background. In wild type Wistar rats this dose was 3 × 40 mg/kg [5,21] and in SD and F344 2 × 60 mg/kg [6]. We screened 768 amplicons in 291 F1 progeny of the 3 × 30 mg/kg group and covered ~70 Mb of sequence. A total of 84 ENU-induced mutations were identified (Table 1). As a result, the overall mutation rate is 1 mutation every 8.29 × 10^5 ^bp, a 1.5-fold increase compared to the highest mutation rate in Msh6-proficient animals.

Only one nest of three pups could be recovered from the single fertile male that was treated with 3 × 35 mg/kg (this animal was sterile in subsequent mating) and one mutation was discovered in the 0.72 Mb that was sequenced (Table 1). 16 F1 progeny from a single male that was treated with 25 mg/kg were screened and in this group 4 mutations were discovered in 3.6 Mb, resulting in a similar mutation rate as observed in the 3 × 30 mg/kg treated group (Table 1).

Although several candidate mutations were found more than once, though at low frequency, analysis of family relationship revealed that these are not due to clonal effects, as in all cases, F1 progeny bearing the same mutations descended from different founders. These polymorphism where thus classified as low frequency SNPs (note that Wistar is an outbred strain) and not included in the results. Unfortunately, mutation density in our study is not high enough to draw any conclusions on the existence of hypersensitive or ENU-resitant regions in the rat genome.

### Mutation frequency changes over time

**Figure 2:**
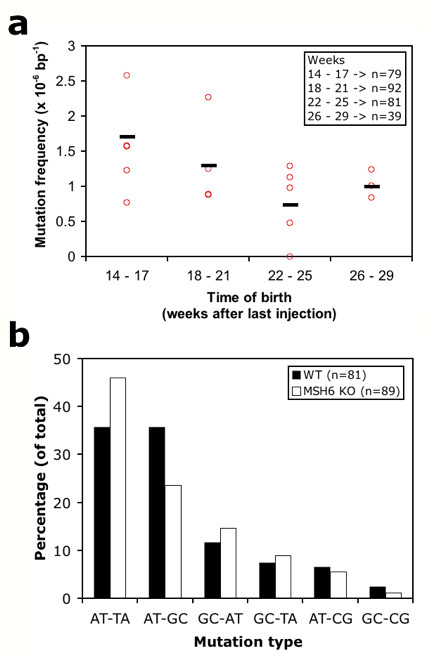
**ENU-induced mutation frequency and spectrum**. (a) The ENU-induced mutation frequency differs in time. Red circles indicate the mutation frequency of the F1 progeny of the 5 different *msh6*^-/-^founders treated with 30 mg ENU/kg. The black stripes indicate the mutation frequency of all the F1 progeny of these founders together for the different time bins. A decrease of mutation frequency is observed in time, which reaches a steady level at 1.0 × 10^-6 ^per bp (1 mutation per 1 Mb). The letter n indicates the number of total F1 screened per time bin. (b) ENU-induced mutation spectrum in Msh6-deficient background (white bars) compared to MMR-proficient animals (black bars).

We analyzed the mutation frequency of the F1 offspring in time bins after mutagenesis of the founders. Offspring from mutant males treated with 3 × 30 mg/kg demonstrate a reduction of mutation frequency in time (Figure 2a). In the 79 animals that were born 14 – 17 weeks after the last ENU injection ~18.7 Mb was analyzed and 32 mutations were discovered, reflecting a mutation frequency of 1.71 × 10^-6 ^per bp and a rate of 1 mutation every 585 kb. Animals that were born 18 – 21 weeks and 22 – 25 weeks after the last ENU injection show a decreased mutation frequency of 1.30 × 10^-6 ^and 0.74 × 10^-6 ^per base pair, respectively. We observed some notable variation in germ line mutation frequencies between the 5 founders. However, this was mainly the result of the differences in litter sizes at the analyzed time points and the variation decreased almost completely when the overall germ line mutation frequency of the individual founders was compared (1.20 × 10^-6 ^± 0.25 × 10^-6^), suggesting only limited inter-animal variability. F1 progeny born later than 25 weeks after the last injection show a slight increase in frequency (~1.0 × 10^-6 ^per bp). However, this observation is based on a relatively low numbers of F1 progeny, derived from a limited number of founders, as the mortality started to increase at this time. The mean survival of ENU-treated *msh6*^-/- ^rats (3 × 30 mg/kg) was 37 ± 3 weeks, which is 23 weeks after the last injection (Figure 1). Notably, all fertile males lived at 37 weeks of age, while the infertile males had mostly died, suggesting a direct relationship between fertility and viability, and probably also the amount of ENU-induced genetic damage.

### ENU-induced mutation spectrum differs in Msh6-deficient background

Interestingly, it was found that ENU induces a different mutation spectrum in an Msh6-deficient background as compared to an MMR-proficient background (Figure 2b). The most common mutations in both wild type as well as Msh6-deficient background were alternations of A-T base pairs (~75%). This is consistent with previous reports and is thought to result from *O*^2^-ethylthymine (*O*^2^-etT) and *O*^4^-ethylthymine (*O*^4^-etT) lesions [17,23]. However, for the A-T pairs a significant increase in A-T to T-A transversions (p = 0.045) and decrease of A-T to G-C transitions (p = 0.019) was observed in the Msh6-deficient background. It has been shown that bypass of *O*^2^-etT lesions induces A-T to T-A transversions, whereas *O*^4^-etT lesions results in A-T to G-C transitions [24,25]. Our data suggests that MutSα preferentially recognizes *O*^2^-etT lesions in the rat.

Due to non-random codon usage and the highly biased composition of translation termination codons, the chance for introducing a premature stopcodon strongly depends on the mutation spectrum. For example, A-T to T-A transversions can introduce a premature stopcodon in 7 out of the total 183 possible changes in the genetic code (excluding the combinations that already code for a stop signal), whereas A-T to G-C transitions will never result in a premature stopcodon. Based on the mutation spectra obtained in wild type and Msh6-deficient backgrounds we calculated the chance of generating a knockout-type allele by introducing a premature stopcodon or mutating splicing donor/acceptor site for the 768 amplicons that were used in our screen. The chance of generating a knockout-type allele was found to be increased by ~21% in the MMR-deficient background (from 4.26% for wild type to 5.16% for the *msh6*^-/- ^mutation spectrum).

### ENU-induced mutations

The 768 amplicons that were used in the mutation screening were designed to cover exons of genes of interest. 84 out of the 89 mutations that were identified reside in protein-coding regions (Table 1). Four mutations (~5%) introduce a premature stopcodon in an open reading frame and are thus most likely to result in a functional knockout of the gene, which corresponds nicely with the expected chance of introducing a premature stopcodon, as discussed above. 64 mutations cause an amino acid change (missense, ~76%) and 16 mutations do not affect protein coding (silent, ~19%), which also correspond with calculated predictions (74% and 22%, respectively). None of the non-coding mutations resided in splicing donor/acceptor sites.

## Discussion

The mutagen ENU can transfer its ethyl group to oxygen or nitrogen radicals present in DNA, which results in lesions that can cause mispairing during replication and eventually give rise to a single base pair substitution [23]. We hypothesized that a deficiency in the repair system that detects or corrects such single base pair damages or mismatches would result in an increased ENU-induced mutation frequency and associated improvement of the target-selected mutagenesis-based knockout procedure. Indeed, deficiency of Msh6 was found to improve the ENU-driven knockout procedure in two ways; 1) it increases the ENU-induced germ line mutation frequency up to 1 mutation every 585 kb and 2) the mutation spectrum is changed and enhances the chance of generating a knockout-type allele by 21%. Cumulatively, this results in a ~2.5-fold increase in knockout efficiency.

The increased mutation frequency was reached at an ENU dose that was 25% lower than in wild types and together with the change in mutation spectrum this argues for the specificity of MMR-deficiency underlying the observed effects. In line with this, an increase in mutagenicity of ENU in MMR-deficient background was also shown in mouse ES cells lacking Msh2 [17]. However, it has to be noted that in zebrafish, which lack Msh6, no difference in ENU-induced mutation frequency has been observed compared to MMR-proficient fish [26]. This apparent contradiction with the results presented here could be explained by the large difference in mutation frequency. In wild type zebrafish, the ENU-induced mutation frequency is about 1 mutation every 100,000 bp, whereas, this frequency in wild type rats is more than 10-fold lower [5,8-12]. Proposedly, the zebrafish mutation load is the maximum that is compatible with viability, a suggestion that is corroborated by comparable maximal mutation frequencies observed in *C. elegans *[13] and Arabidopsis [14]. Our results suggest that the lower ENU-induced mutation frequencies in rodents can at least partially be explained by more efficient mismatch repair in the testis that counteracts mutagenicity, and is less likely due to increased sensitivity to genotoxic damage in general. The decline of the mutation frequency in time that is observed in this study, however, could indicate the presence of genotoxic effect. Initial depletion of spermatogonial stem cells could be due to apoptosis induced by ENU damage – a mechanism that is presumed to be underlying the sterility effect at higher doses. Selective repopulation of the testis by the most viable stem cells with presumably the lowest amount of genotoxic damage, decreases the apparent mutation frequency (and potentially increases the change for clonal progeny). Our results indicate that the target-selected mutagenesis works most efficient for F1 progeny resulting from matings at about 10 – 14 weeks after the last ENU injection. However, it should be kept in mind that only F1 animals, generated after one full cycle of spermatogenesis (in rat and mouse about 60 to 70 days) after mutagenesis, should be screened in order to prevent retrieval of chimaeras. Such chimaeras can be induced by ethyl-DNA adducts in the fertilized oocyte that originate from mutagenized post-meiotic sperm cells and which could result in fixation of mutation in different lineages during embryonic development.

**Figure 3:**
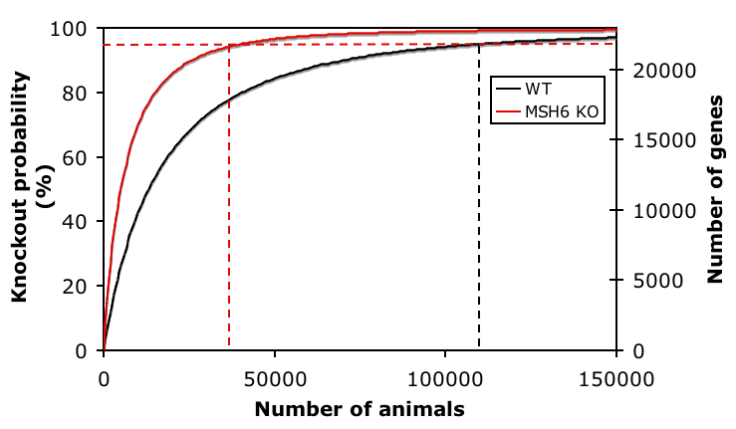
**Probability of gene knockouts in Msh6-deficient and wild type rats**. The chance to retrieve a knockout for any given gene and the total number of genes that will be knocked out when all genes would be screened for mutations is plotted as a function of the number of mutant F1 animals for wild type (WT, 40 mg ENU/kg, black line) and MMR-deficient (Msh6^-/-^, 30 mg ENU/kg, red line) rats. The red dashed lines show the number of animals needed to knockout 95% of all genes and any given gene with 95% chance. The use of a Msh6-deficient background reduces the number of animals ~2.5-fold.

Besides the ENU mutagenesis approach, other gene targeting technologies are being developed for the rat, which include nuclear transfer, although to date no genetic modification has been reported, and knockdown by RNA interference (reviewed in [27]). Recently, transposon-tagged mutagenesis [28] was successfully applied to the rat [29] as well and although this technique is currently less amendable for scaling, it should be considered as highly complementary to the existing ENU-based efforts. The ENU-driven target-selected mutagenesis approach has already been used successfully for generating a variety of novel rat knockout models [5,6,16] and with the improvements described here, this approach does provide realistic technological requirements for screens on a genome-wide scale. In a wild type background, ~110,000 F1 rats would be needed to knockout any given gene with 95% probability (Figure 3). This number is reduced to 40,000 in the MMR-deficient background. When reasoning the other way around, 40,000 F1 rats will contain knockouts of 95% of all rat genes and when considering 'only' 5,000 F1 rats, knockout alleles for ~50% of all the rat genes will be present. It should be said, however, that to identify these knockout alleles, the complete ORFeome would have to be screened. Although existing technologies are not suited for this, emerging massively parallel sequencing technologies [30], and microarray-based enrichment procedures [31,32] provide promising avenues in this direction. Keeping this in mind, archiving frozen sperm samples of mutant F1 animals, which can be recovered by intracytoplasmic sperm injection (ICSI) [33,34] becomes very attractive.

The identification of a wide range of potentially interesting missense mutations as well as the retrieval of four novel candidate knockout models resulting from the introduction of premature stopcodons by screening only a small set of about 300 animals illustrates the power of the approach. Furthermore, the ENU-based approach has the potential to generate allelic series (multiple mutations in the same gene) in different animals, which can facilitate the identification of novel gene functions. For example, hyper- and hypomorphic mutations provide information related to gene dosage effects and residues important for specific protein interactions or enzymatic functions can be identified.

Currently, the 4 knockouts and 45 selected missense mutations are being crossed to the next generation and bred to homozygosity for subsequent phenotypic analysis. As homozygous Msh6-deficiency, which could occur in later generations, would result in further accumulation of novel mutations, this outcross is also used to eliminate the mutant *msh6 *allele from the genetic background by genotype-assisted breeding. In addition, further outcrossing to wild type background should be performed and littermates should be included as control animals in phenotypic characterization experiments to minimize confounding effects of background mutations. Although such effects should never be ignored, estimates do indicate that this potential problem should not be exaggerated [35], especially if outcrossing is combined with marker-assisted selection for which the outbred Wistar background is well-suited.

## Conclusion

We have significantly improved the target-selected mutagenesis gene knockout technology for rats by taking advantage of deficiency of specific MMR function. As mutagen-driven target-selected mutagenesis approaches have become popular in organisms for which no ES cell-based techniques are available, such as medaka [36], but also plants like rice [37], Lotus [38] and maize [39], the improvements described here may equally well be applied to other species for optimization of gene knockout efficiency.

## Methods

### Animals and ENU mutagenesis protocol

All experiments were approved by the Animal Care Committee of the Royal Dutch Academy of Sciences according to the Dutch legal ethical guidelines. Experiments were designed to minimize the number of required animals and their suffering. Male Msh6 knockout rats (*Msh6*^*1Hubr*^) of 12 weeks of age were given three weekly intraperitoneal injections of 1, 5, 10, 20, 25, 30 and 35 mg ENU/kg bodyweight. Preparation of ENU (Isopac; Sigma) was done as described [5]. Injected males were monitored for fertility by breeding with one or two females, starting 3 weeks after the last mutagenesis treatment. Pups from these early matings were counted, but not analyzed. Ten weeks after the last injection, fertile males of the highest-dosed fertile group were kept on a weekly breeding scheme with two females to produce F1 progeny for mutational analysis.

Animals were housed under standard conditions in groups of two to three per cage per gender under controlled experimental conditions (12-h light/dark cycle, 21 ± 1°C, 60% relative humidity, food and water *ad libitum*).

### Genomic DNA isolation, PCR and sequencing conditions

At two weeks of age F1 progeny were uniquely tagged by ear clipping and the resulting tissue fragments were lysed as described [5], followed by phenol/chloroform (1:1, vol/vol) extraction. DNA was precipitated by adding 300 μl isopropanol, mixing and centrifuging for 20 min, at 21,000 *g *at 4°C. The supernatant was discarded and pellets were washed with 70% ethanol and dissolved in 500 μl 10 mM Tris-HCl (pH 8.0). 768 pre-selected amplicons were amplified using a nested PCR setup as described [5] with the following modifications. The first PCR reaction was carried out in 2 × 384 wells plates per F1 animal sample, in a total volume of 5 μl, and every well contained a unique set of primers (0.2 μM of each). After thermocycling, the PCR1 reactions were diluted with 20 μl H_2_O and 1 μl was used as template for the second round of PCR, which was carried out in the same 2 × 384 wells format as the first PCR, however, with different sets of primers which are internal to the first set (nested). Sequencing reactions contained 0.1 μl BigDye (v3.1; Applied Biosystems), 1.9 μl BigDye Dilution Buffer (Applied Biosystems) and 0.4 μM of the forward primers used for the PCR2 reaction in a total volume of 5 μl. After thermocycling purified sequencing products were analyzed on a 96-capillary 3730XL DNA analyzer (Applied Biosystems), using the standard RapidSeq protocol on 36 cm array. Sequences were analyzed for the presence of heterozygous mutations using PolyPhred [22] and in-house developed software. All candidate mutations were verified in independent PCR and sequencing reactions.

### Project management and primer design using LIMSTILL

All resequencing projects were designed and managed using LIMSTILL, LIMS for Induced Mutations by Sequencing and TILLing (V.G., E.C., unpublished). This web-based publicly accessible information system  was used to generate projects and visualize gene structures based on Ensembl genome data, the design of PCR primers, entry, archiving and primary interpretation of mutations. The primer design application within LIMSTILL is Primer3-based [40] and parameters are set to design primers with an optimal melting temperature of 58°C.

### Knockout probability calculation

Calculation of the frequency of introducing a premature stopcodon with different mutation spectra is an integrated feature of LIMSTILL . The chance of generating a knockout-type allele was calculated for all 768 working amplicons by comparing the probability of nonsense mutations and splicing donor/acceptor mutations divided by all changes in coding sequence plus splicing donor/acceptor sites (first two and last two nucleotides of each intron), all corrected for the wild type and mutant mutation spectra. To calculate the knockout probability both the mutation frequency and corresponding spectrum were taken into account. Rat Ensembl Build 47.34q was downloaded from ftp.ensembl.org. For every gene the longest transcript (22,959 in total) was used to calculate the total number of coding nucleotides. Wistar animals treated with 40 mg/kg ENU represent the most optimal wild type group and were compared with F1 progeny from *msh6*^-/- ^animals that was born 14 – 17 weeks after the last ENU injection.

### Statistical analysis

For calculating statistical differences in the mutation spectrum data the chi-squared test was used. P < 0.05 was considered to be statistically significant.

## Abbreviations

ENU: N-ethyl-N-nitrosourea; MMR: mismatch repair; IDL: insertion/deletion loop; MSI: microsatellite instability; ORFeome: open-reading-frame-eome.

## Authors' contributions

RB was involved in study design, data collection and analysis, interpretation of results and manuscript preparation. PT, MV and HR were involved in data collection. IJN was involved in bioinformatics analyses. VG was involved in bioinformatics analyses and interpretation of results. EC was involved study design, data analysis, interpretation of results and manuscript preparation.
